# Lipid Composition of Nanocarriers Shapes Interactions of Cyclic Antimicrobial Peptide Gramicidin S with Their Membranes

**DOI:** 10.3390/ijms26146946

**Published:** 2025-07-19

**Authors:** Volodymyr Berest, Larysa Sichevska, Olga Gorobchenko, Ihor Perepelytsia, Galyna Bozhok, Oleksii Skorokhod

**Affiliations:** 1 Department of Molecular and Medical Biophysics, School of Radio Physics, Biomedical Electronics and Computer Systems, V. N. Karazin Kharkiv National University, 4 Svobody Sq, 61022 Kharkiv, Ukraine; sichevska@karazin.ua (L.S.); gorobchenko@karazin.ua (O.G.);; 2 Department of Cryoendocrinology, Institute for Problems of Cryobiology and Cryomedicine of the NAS of Ukraine, 23 Pereyaslavska St., 61016 Kharkiv, Ukraine; 3 Department of Life Sciences and Systems Biology, University of Turin, 10123 Turin, Italy

**Keywords:** antimicrobial peptides, gramicidin S, lipid membranes, delivery systems, nanocarriers

## Abstract

Gramicidin S (GS), an antimicrobial peptide (AMP), exhibits broad-spectrum activity against bacteria and cancer cells but is limited in clinical use due to its cytotoxicity toward eukaryotic cells. Lipid-based delivery systems may overcome this limitation; in this study, we proposed and tested simple and promising lipid formulations, including dipalmitoylphosphatidylcholine (DPPC), cardiolipin (CL), and cholesterol (CHOL). We evaluated the interactions of these lipid membranes with GS by assessing membrane fluidity, dielectric permittivity, dielectric losses, dielectric relaxation frequency, and static dielectric constant. Among these, membrane fluidity and dielectric permittivity were the most sensitive to GS, showing significant changes in the formulation containing 90 mol% DPPC and 10 mol% CHOL when exposed to 20 μM GS. Notably, although membrane fluidity changed in a dose-dependent manner following GS binding, the liposomes still supported relatively high GS concentrations—up to 80 μM—which is important for future high-dose GS applications. Additionally, we performed preliminary cytotoxicity tests comparing free GS with liposome-carried GS using the tested lipid compositions and observed a significant reduction in GS-associated toxicity on L929 cell line. This study provides new insights into GS–membrane interactions and supports the rational design of AMP nanocarriers for biomedical applications.

## 1. Introduction

Antimicrobial peptides (AMPs) have gained increasing attention as promising therapeutic agents due to their broad-spectrum activity against bacteria, fungi, and even cancer cells, along with a reduced propensity for resistance development compared to classical antibiotics [1,2]. Among them, gramicidin S (GS) stands out as a cyclic decapeptide from Bacillus brevis with potent antimicrobial and antitumor properties [3,4,5]. Its amphipathic structure facilitates integration into lipid bilayers, resulting in membrane disruption and cellular death. Despite its efficacy, the therapeutic use of gramicidin S has been limited due to its cytotoxicity toward eukaryotic cells. Therefore, the development of GS delivery systems (such as lipidic nanoparticles) that enhance GS selectivity and reduce side effects is important, as understanding GS interactions with biological lipid membranes, which constitute these nanoparticles, is crucial.

To investigate GS–membrane interactions, model membranes could be used, such as lipid vesicles or liposomes with defined lipid compositions. The selection of lipid components for the creation of lipidic nanoparticles is pivotal, as it aims, on one hand, to mimic key biophysical features of natural membranes, and on the other hand, to be simple while maintaining integrity to support the carried medicine. Eukaryotic cell membranes are primarily composed of phosphatidylcholine (PC), sphingomyelin, and cholesterol (CHOL), which contribute to membrane fluidity and structural integrity. Additionally, phosphatidylethanolamine (PE) and phosphatidylserine (PS) are present in variable amounts, playing roles in membrane curvature, signaling, and interactions with proteins. CHOL, in particular, plays a fundamental role in modulating membrane fluidity and integrity, and is largely absent in prokaryotic membranes. Bacterial membranes are rich in phosphatidylglycerol (PG), cardiolipin (CL), and PE, and are generally more negatively charged, which contributes to their increased susceptibility to cationic AMPs like GS.

What is known about GS interaction with lipid membranes is that, in model membranes, GS binds more strongly to negatively charged lipids than to zwitterionic or uncharged phospho- and glycolipids. GS incorporation into the bilayer leads to slight membrane thinning and preferential localization near glycerol residues, where it interacts with polar head groups and the upper regions of lipid hydrocarbon chains, likely due to the presence of the positively charged ornithine residue in GS [6,7]. GS binding disrupts liquid-crystalline bilayers in a dose-dependent manner, increasing non-specific permeability in both model and biological membranes [6,8], a key mechanism underlying its antimicrobial action. At low concentrations, GS induces bilayer thinning, as mentioned above, while at higher concentrations, it can promote the formation of inverted non-lamellar cubic phases in phospholipid dispersions [9]. Differential scanning calorimetry (DSC) has identified GS sensitivity to the phase state of model lipid membranes, showing that the peptide localizes at the polar–nonpolar interface of liquid-crystalline layers. Its primary interactions occur with the polar head groups of lipids, while secondary interactions involve hydrophobic lipid tails [6,10].

The effect of cholesterol on different lipid bilayers has been widely studied [11]. If cholesterol does not reach exaggerated concentrations that break lipid bilayers, a relatively high cholesterol content in the membrane contributes to increased elasticity [12]. Excess cholesterol weakens the connections between layers, and the bilayer behaves as an unlinked system. This instability in the lipid bilayer promotes the rapid redistribution of cholesterol within the bilayer, leading to lipid asymmetry [11]. Regarding GS binding and distribution in cholesterol-containing lipid bilayers, the modification of phosphatidylcholine vesicle bilayers with cholesterol molecules does not directly affect the conformation and biological activity of the GS molecule. However, CHOL facilitates the integration of GS molecules into a more polar region of the bilayer and weakens the interaction of the GS with phospholipid membranes [13]. FTIR spectroscopy data suggest that the presence of cholesterol leads to greater exclusion of GS molecules in dipalmitoylphosphatidylcholine (DPPC) membranes [13]. This finding is unexpected, as cholesterol is well known for expanding and disrupting phospholipid layers in the gel state, and was therefore expected to facilitate GS binding. Experimentally, the rigid fused ring structure of the cholesterol molecule, along with its predominantly hydrophobic and minimally hydrated interactions with polar head groups and fatty acyl chains in the phospholipid bilayer, plausibly impedes the incorporation of GS molecules. Further studies are requested using membranes composed of non-DPPC lipids [13]. It is also known that cholesterol alters the mechanical strength of 1,2-dioleoyl-sn-glycero-3-phosphocholine (DOPC) bilayer. In its pure form, the DOPC bilayer functions as a single connected system, while CHOL induces localized disruptions, as observed through atomic force microscopy [14]. This suggests that GS may integrate more readily into CHOL-containing liposomes and remain within them for drug delivery applications. In summary, CHOL appears to be a promising component in lipid nanocarrier membranes for GS. However, further studies are needed to better understand its interaction with GS within the given lipid environment.

In this study, we tested selected model lipid bilayers of different compositions with CHOL for interaction with GS. This approach enables further comparative analysis of GS interactions with carrier membranes and final targeted membranes, providing deeper insight into GS selectivity and mechanism of action. To test the GS biocompatibility with live cells and compare the GS cytotoxicity with lipid nanoparticles carried GS, we employed cultured L929 cell line as a model of subcutaneous-derived cells. Connective tissue cells are not only physiologically relevant as the first line of defense against pathogens but also represent a key barrier for certain drug delivery applications. Therefore, they could serve as in vitro platforms to assess both biological activity and the safety of AMP-loaded nanocarriers.

Taken together, this study explores the biophysical interactions of gramicidin S with membranes of varying lipid compositions and CHOL, with the overarching goal of optimizing AMP delivery strategies for biomedical applications.

## 2. Results

The sensitivity of liposome membranes to GS in the concentration range from 1 μM to 80 μM was studied using the fluorescence spectroscopy method. The effect of GS on the redistribution of surface charges and on the packing density of fatty acid residues in the hydrophobic region of model membranes of different phospholipid composition was studied using fluorescent probes pyrene and 8-anilino-1-naphthalenesulfonate (ANS). It is known that the lipophilic probe pyrene is effectively redistributed in the hydrophobic region of the lipid bilayer and responds to the packing density of the hydrocarbon tails of phospholipids by the degree of formation of excimer forms [15].

**Figure 1:**
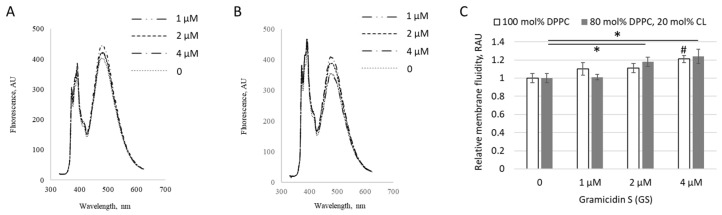
GS-induced changes in liposome fluidity. Fluorescence spectra of excimerized pyrene, reflecting membrane fluidity, are shown for (**A**) liposomes composed entirely of dipalmitoylphosphatidylcholine (DPPC) and (**B**) liposomes containing 80 mol% DPPC and 20 mol% CL, without GS (dotted line) and with GS added at concentrations of 1–4 µM (dashed lines). (**C**) Membrane fluidity as reflection of pyrene excimerization signal, expressed in relative arbitrary units (RAU). Means ± SD for 3 independent preparations and significance of difference vs. untreated liposomes by * for *p* < 0.05 are reported for liposome composition with 20 mol% CL (gray columns). The “#” indicates the significance of differences compared to GS-untreated liposomes (left column indicated by 0) with 100 mol% DPPC composition (white columns).

At low concentrations (1–4 μM), GS incorporated into liposomes altered the extent of pyrene excimer formation within membranes, as shown for both pure DPPC liposomes (Figure 1A) and liposomes containing 20 mol% of CL (Figure 1B), indicating perturbation of the hydrophobic region of the lipid bilayer by GS molecules. The changes were more pronounced in liposomes containing CL, increasing significantly by 24 ± 8%, likely due to the negative charges of CL, which enhanced interactions with the positively charged ornithine groups in GS.

**Figure 2:**
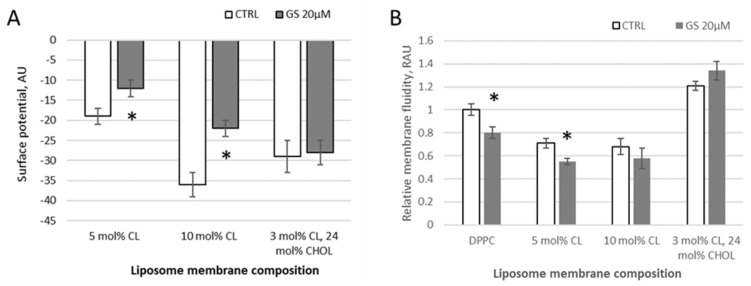
Changes in liposome membrane surface potential and fluidity with different membrane compositions induced by GS. (**A**) Changes in the surface potential derived from ANS probe signal and reported in arbitrary units (AU) and (**B**) changes in membrane fluidity, derived from pyrene signal and reported in relative arbitrary units (RAU) are shown for liposomes composed of 100% DPPC (left two columns); 95 mol% of DPPC and 5 mol% CL (indicated in figure as 5 mol% CL), 90 mol% of DPPC and 10 mol% CL, and 73 mol% of DPPC, 3 mol% CL, and 24 mol% CHOL (right-side two columns). Control untreated liposomes (white columns, CTRL) were compared to 20 μM GS-treated liposomes (gray columns), means ± SD for three independent preparations are plotted and the differences are indicated by “*” for *p* < 0.05.

Using a water-soluble anionic fluorescent probe ANS, the membrane surface of lipid vesicles was monitored and the surface potential of model membranes in the presence of GS was calculated. With increasing negative surface charge of the membrane by CL presence, the fluorescence intensity of ANS decreases in observed samples (DPPC with 5 mol% of CL, with 10 mol% of CL, and liposomes from a lipid mixture of DPPC with 3 mol% of CL and 24 mol% of CHOL) due to a decrease in the number of positively charged probe sorption centers (Figure 2A). According to the data of fluorescence spectroscopy, calculations of the surface potential of model membranes were made. When 20 μM GS was bound to the membranes, significant changes of 37 ± 15% (surface potential absolute value was changed from −19.1 to −12.2 units) and 39 ± 12% (from −36.2 to 22.1 units) in the surface potential were observed in liposomal samples with a cardiolipin content of 5% and 10%, respectively (Figure 2A). At these conditions, GS appears to perturb the packing density of lipids.

In samples of neutral (pure DPPC) and charged (with CL) liposomes, the addition of 20 μM GS tends to reduce the degree of pyrene excimerization and thus membrane fluidity at limited concentrations of CL. Initially, in pure DPPC, the pyrene-detected fluidity decreased by 20 ± 5% and in membranes with 5 mol% CL, it decreased significantly by 24 ± 7%, whereas in those with 10 mol% CL, the decrease was non-significant, only by 14 ± 10% (Figure 2B). This reduction in the pyrene signal, corresponding to an increase in membrane micro-viscosity, was similar to the effect observed at lower GS concentrations (1–4 μM) (Figure 1). The inclusion of CHOL in the DPPC/CL liposomes modifies the bilayer rigidity, showing increased pyrene signal, and the addition of GS at a concentration of 20 μM leads to an additional increase in the pyrene signal in DPPC/CL membranes (Figure 2B). Note that the ethanol, in which the stock GS was dissolved, did not affect the measured parameters when diluted according to GS treatment concentrations.

**Figure 3:**
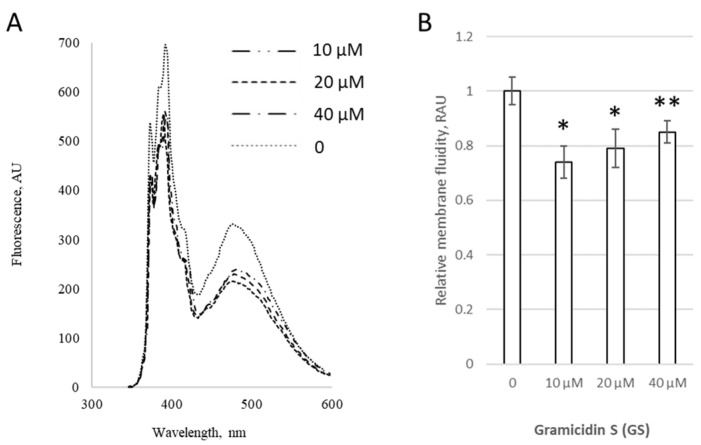
GS changes the membrane fluidity in liposomes with a fixed CHOL content. (**A**) Fluorescence spectra of pyrene in liposomal membranes containing 10 mol% CHOL with the addition of 0–40 μM of GS. (**B**) Relative membrane fluidity, derived from pyrene signal and reported in relative arbitrary units (RAU) are shown for liposomes incubated with 0–40 μM of GS. Control untreated liposomes (first column) were compared to GS-treated liposomes, means ± SD for three independent preparations are plotted and the significance of differences are indicated by “*” for *p* < 0.05 and by “**” for *p* < 0.07.

**Figure 4:**
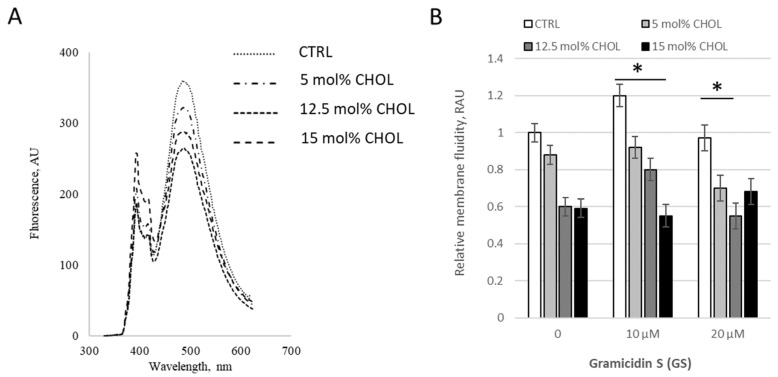
GS changes the membrane fluidity in liposomes with varying CHOL content. (**A**) Fluorescence spectra of pyrene in liposomal membranes containing cholesterol under the addition of GS at a concentration of 20 μM. (**B**) Relative membrane fluidity, derived from pyrene signal and reported in relative arbitrary units (RAU) are shown for liposomes with different CHOL composition incubated with 0, 10 and 20 μM of GS. Results for control DPPC liposomes (white columns) were compared to CHOL contained liposomes, means ± SD for three independent preparations are plotted and the differences are indicated by “*” for *p* < 0.05.

**Figure 5:**
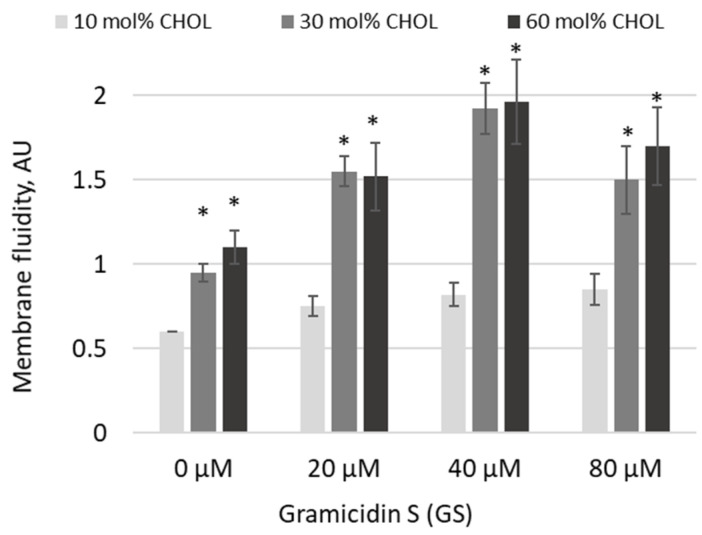
GS increased membrane fluidity at high treatment concentrations in membranes with elevated CHOL content. DPPC membranes containing 10–60 mol% CHOL were treated with varying concentrations of GS, and membrane fluidity was assessed using pyrene fluorescence following excimer formation, reported in arbitrary units (AU). Means ± SD for three independent preparations are plotted and the differences are indicated by “*” for *p* < 0.05.

Given the results with GS reported in Figure 1, and considering the theoretical significance of CHOL presence in GS interaction with lipid membranes, we next examined the dependency of various GS and CHOL concentrations on GS effects in PC membranes (Figure 3, Figure 4 and Figure 5).

Initially, we assessed the modifications induced by 0–40 μM GS in PC membranes with a fixed content of 10 mol% CHOL (Figure 3). GS treatment at 10 μM initially significantly decreased the pyrene signal by 26 ± 6%, but at higher GS concentrations, such as 40 μM, the signal increased by 11% compared to 10 μM GS; however, it remained lower than the control, with statistical significance at *p* < 0.07 (Figure 3B), highlighting the importance of accounting for GS concentration.

We then tested membrane viscosity at fixed GS treatment concentration 20 μM with varying CHOL content (Figure 4). When CHOL content increased in “low concentration range” from 0 to 15%, moderate GS concentration 10 μM led to a decrease in the pyrene signal, resulting in an increase in bilayer microviscosity (Figure 4B), but at the combination of highest concentrations (15% of CHOL and 20 μM of GS) inverted the tendency, the pyrene signal is increased (Figure 4B, last black column) and the membranes started to be more fluid. The increase (last black column vs. gray column in Figure 4B) was 24 ± 14%, with a difference significance of *p* = 0.088, which is not marked in the Figure.

Based on trends observed at high GS and CHOL concentrations, in the next experiments, we increased both GS treatment concentrations and CHOL content (Figure 5). Indeed, pyrene excimerization significantly increased as CHOL concentrations rose from 10 mol% to 30 and 60 mol% (Figure 5, significances of differences are indicated vs. first light gray column). In 30 and 60 mol% CHOL-content membranes, the addition of GS at increasing concentrations led to a further rise in the pyrene signal at 20 and 40 μM GS, while at 80 μM the signal remained elevated but was not the highest observed (Figure 5). The maximum increase in membrane fluidity was observed with a combination of 30 and 60 mol% CHOL and 40 μM GS (Figure 5).

**Figure 6:**
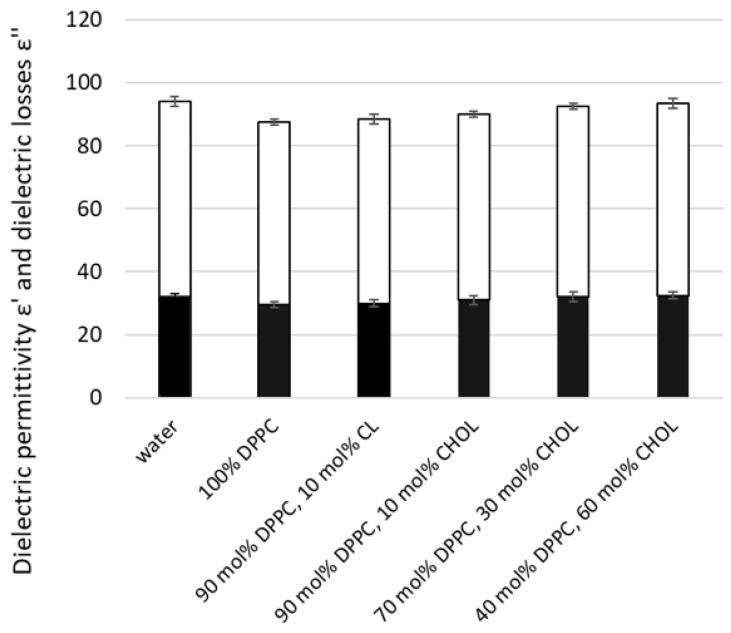
Dielectric permittivity ε′ and dielectric losses ε″ of lipid nanocariers’ suspensions with different compositions compared to water. Lipid nanocarriers were created as described in the Methods section, using only DPPC 100%; 90 mol% DPPC and 10 mol% CL; 90 mol% DPPC and 10 mol% CHOL; 70 mol% DPPC and 30 mol% CHOL; 40 mol% DPPC and 60 mol% CHOL. Measurements were conducted using the microwave dielectrometry method at a frequency of 9.2 GHz and ε′ is reported in upper white columns and ε″ reported in lower black columns. Means ± SD for five-six measurements of one representative preparation are plotted.

Another parameter of lipid membranes, dielectric permittivity and dielectric losses of liposomes, was measured using the microwave dielectrometry method. Initially, we tested liposomes with different compositions, including basal 100% DPPC liposomes, followed by liposomes containing various concentrations of CL and CHOL (Figure 6). The lowest dielectric permittivity and losses values were characteristic of PC liposomes. The addition of CL or CHOL slightly increases the dielectric parameters of liposome suspensions, with a greater effect as the CHOL fraction rises. This indicates an increase in the amount of free water in the suspensions, which may result from liposome dehydration.

**Figure 7:**
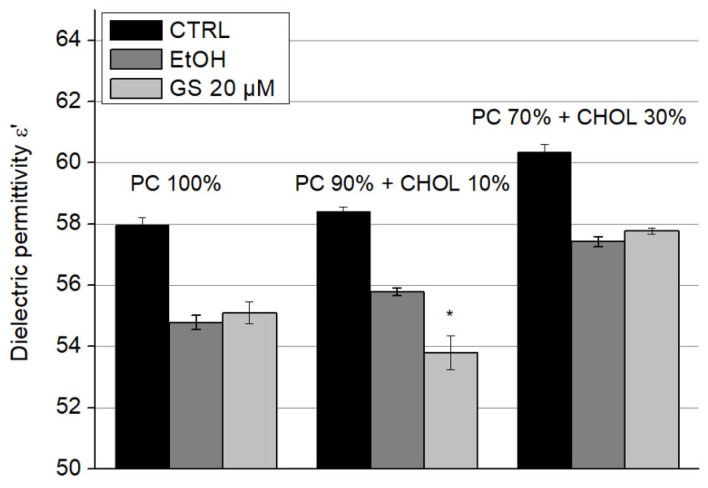
GS decreased the dielectric permittivity ε′ of liposomes with 10 mol% of CHOL. Effect of GS on the dielectric permittivity of liposome suspensions at a frequency of 9.2 GHz was measured for liposome compositions: 100% DPPC (left three columns), 90 mol% DPPC and 10 mol% CHOL (central three columns), and 70 mol% DPPC and 30 mol% CHOL (right side three columns). Means ± SD for five-six measurements of one representative preparation are plotted and the differences are indicated by “*” for *p* < 0.05.

**Figure 8:**
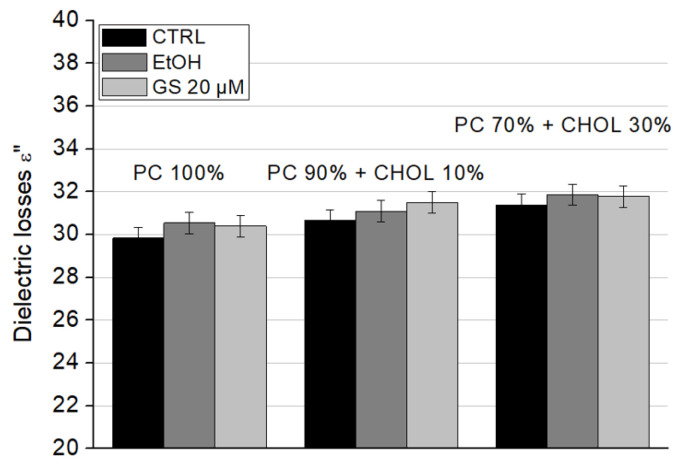
Effect of GS on the dielectric losses ε″ of liposome suspensions at a frequency of 9.2 GHz was measured for liposome compositions: 100% DPPC (left three columns), 90 mol% DPPC and 10 mol% CHOL (central three columns), and 70 mol% DPPC and 30 mol% CHOL (right side three columns). Means ± SD for five-six measurements of one representative preparation are plotted.

The dielectric permittivity ε′ of liposome suspensions upon the addition of GS is presented in Figure 7 and dielectric losses (ε″) in Figure 8. Since these parameters were sensitive to ethanol, in which GS was dissolved—even at low concentrations—its influence on the dielectric properties of the samples was accounted for by separately adding ethanol to the liposome suspensions at concentrations corresponding to those of diluted GS. A significant decrease of 5.5 ± 1% in dielectric permittivity (ε′) due to GS was observed only in liposomes containing 10 mol% CHOL (Figure 7).

The dielectric losses (ε″) showed a slight increase due to GS; however, this change was not significant for any liposome type (Figure 8).

**Figure 9:**
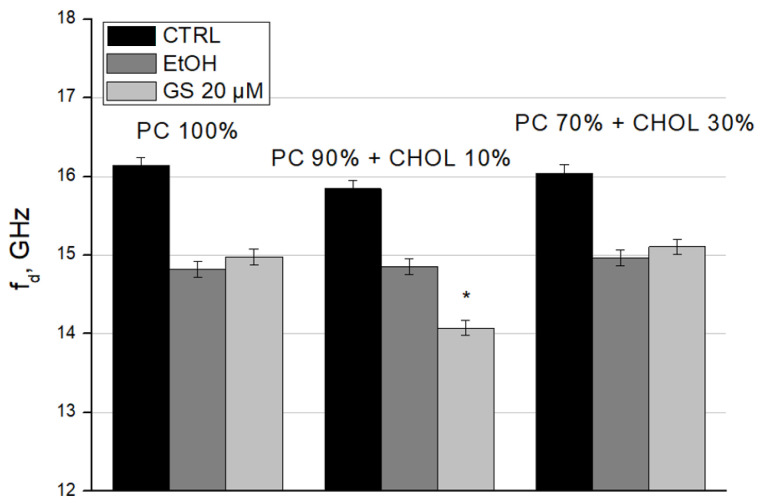
Dielectric relaxation frequency (f_d_) of liposome suspensions was measured for liposome compositions: 100% DPPC (left three columns), 90 mol% DPPC and 10 mol% CHOL (central three columns), and 70 mol% DPPC and 30 mol% CHOL (right side three columns). Means ± SD for five-six measurements of one representative preparation are plotted and the differences are indicated by “*” for *p* < 0.05.

**Figure 10:**
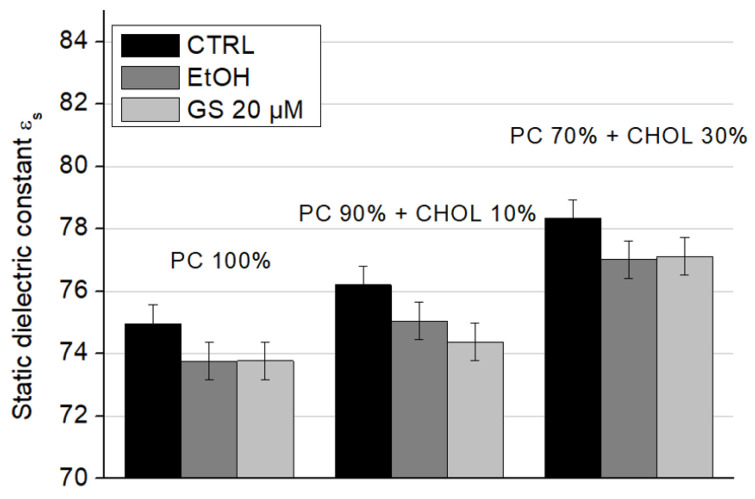
Static dielectric constant (ε_s_) values of liposome suspensions measured at a frequency of 9.2 GHz for different liposome compositions: 100% DPPC (left three columns), 90 mol% DPPC and 10 mol% CHOL (central three columns), and 70 mol% DPPC and 30 mol% CHOL (right side three columns). Means ± SD for five-six measurements of one representative preparation are plotted.

To complete the characterization of GS interaction with liposomes, we calculated the relaxation frequency (f_d_) (Figure 9) and the static dielectric constant (ε_s_) (Figure 10) of liposome suspensions using the Debye equations. We observed the decrease in f_d_ in GS-treated liposomes for liposome compositions with 10 mol% CHOL (Figure 9). Such changes in dielectric relaxation indicate the ordering in the bulk water structure within the sample, resulting in decreased mobility of water dipoles (Figure 9).

When cholesterol is added to the composition of model lipid membranes, an increase in the static dielectric constant values is observed: the presence of 10 mol% CHOL increases ε_s_ significantly by 1.6 units, and the presence of 30 mol% CHOL by 3.5 units (Figure 10, white columns). The addition of ethanol and GS does not change this trend due to CHOL. However, ethanol itself reduces the static dielectric constant approximately by 1–2 units in all conditions. At a concentration of 20 µM, GS slightly decreased the static dielectric constant of liposomes containing 10 mol% CHOL compared to the ethanol control (Figure 10, central columns). These changes may be a consequence of an increase in the amount of lipid-bound water due to a change in the physical properties of the lipid bilayer due to GS.

**Figure 11:**
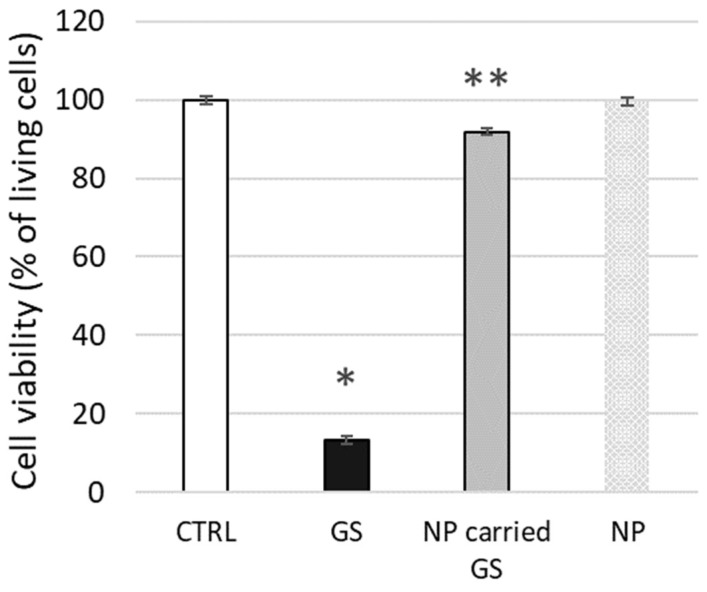
GS carried by nanoparticles reduces the toxicity of free GS. L929 model cells were treated with free GS at 25 µg/mL concentration, with nanoparticle-carried GS (NP-carried GS) or control free nanoparticles (NP), and cell viability was measured using the fluorescein diacetate-ethidium bromide (FDA-EB) viability test. The significance of differences between untreated cells (CTRL) and GS-treated cells is *p* < 0.01 and indicated by “*”, while the difference between GS and NP-carried GS is *p* < 0.03 and indicated by “**”.

To conclude our tests on live biological membranes, we evaluated the potential cytotoxic effect on L929 cells of GS encapsulated in liposomes composed of 10 mol% CHOL, as tested above using biophysical methods. The toxicity of free GS at 25 µg/mL (approx. 20 µM) concentration was relatively high, with 13.3 ± 2.6% cell viability, whereas liposome-encapsulated GS was significantly less toxic, maintaining 91.9 ± 5.7% cell viability (Figure 11). The confluence of the cell monolayer was strongly affected by free GS, while the effect was minimal after cell treatment by encapsulated GS at the same concentration.

## 3. Discussion

Antimicrobial peptides hold potential as a possible alternative, or complement, to conventional antibiotics but new, safe, and efficient means are needed for formulation and administration of the peptides. Non-specific toxicity of some AMPs, including GS, require a delivery transport system. Some examples of proposed delivery approaches are liposomes [6,16], the polyethylene glycol-stabilized lipid disks [17] and others. Interesting applications of antimicrobial peptides (AMPs) have recently been growing in the agritech field for plant pathogen and pest treatment [18].

Dipalmitoylphosphatidylcholine (DPPC) is a key component of lipid bilayers, forming stable lamellar structures. Cholesterol decreases membrane fluidity, enhances stability, and reduces permeability by positioning its hydroxyl group toward the aqueous phase and embedding its ring structure among phospholipid tails. Cardiolipin, with two anionic phosphoric acid residues, has hydrophobic tails mainly composed of C-18 chains with unsaturated bonds. Varying the concentration of cardiolipin during the formation of liposomes allows modeling the level of their potential at the hydrodynamic shear limit (zeta potential) [19]. Liposomes composed of DPPC, CL, and CHOL naturally emerge as promising nanocarriers.

The cardiolipin concentrations examined in this study range from 5–10% in Figure 2 and Figure 6, to 20% in Figure 1. These values were selected based on reported physiological concentrations in various biological membranes. It has been described that cardiolipin constitutes approximately 15–20% of the inner mitochondrial membrane and about 1–5% of the outer mitochondrial membrane lipid content [20,21,22]. Some membrane regions can contain up to 30% cardiolipin and are associated with mitochondrial membrane disorders, apoptotic regulation, and other specific physiological conditions [23]. Cardiolipin is also present in certain bacterial membranes, where it supports the function of essential membrane proteins and can constitute approximately 1–10% of the polar lipids in the inner membrane [21,24]. Cardiolipin has also been used in experimental membrane models for various biophysical studies [25,26], with the aim of simulating surface membrane potential. In summary, based on this evidence, cardiolipin concentrations of up to 20 mol% were chosen for this study.

The antibacterial activity of GS has been reported in the range of 3–12.5 μg/mL, corresponding to approximately 3–10 μM [27,28]. Therefore, the GS concentrations studied in this paper (1–4 μM in Figure 1) fall within the average therapeutic range, while the most interesting 20 μM and extreme 80 μM concentrations were selected to represent a higher dose relevant for delivery systems. As in various preliminary experiments and previous studies involving the lipophilic binding of GS to membranes [6,29], the proportion of unbound GS was also determined as additional information in some of the experiments reported in this paper. The results showed that the majority of GS was bound to both experimental and natural membranes.

Previous studies on GS, CHOL, and lipid interactions in model liposomes have suggested that GS influences the membrane properties [5,6,10]. The lipid formulations proposed and tested in this study, including PC, CL, and CHOL components, are simple and promising. The interaction of these membranes with GS, as well as parameters such as membrane fluidity, dielectric permittivity, dielectric losses, dielectric relaxation frequency, and static dielectric constant, has not been studied previously. Additionally, we tested, for the first time, the effect of GS compared to liposome-carried GS on L929 fibroblasts.

Applying fluorescence spectroscopy, we observed that membrane fluidity generally changed after GS binding to membranes. Some lipid compositions were more sensitive, while others were less sensitive to GS. For example, the presence of CL, with its negative charge, influenced the overall membrane interaction with GS, which contains a positively charged ornithine group. CHOL presence, highly relevant for carrier liposomes, strongly influenced the effects of GS on membrane fluidity (Figure 1, Figure 2, Figure 3, Figure 4 and Figure 5). Less disturbed fluidity was observed with CHOL at 10 mol% (light gray columns in Figure 5) for all tested GS concentrations, up to 80 µM. An increase in CHOL presence, combined with high GS concentrations, led to major changes in membrane fluidity.

Interestingly, in Figure 5, relatively high concentrations of GS—when necessary for therapeutic formulations—could be supported by liposomes, as the changes in fluidity were similar for 20, 40, and 80 µM of GS.

According to the data of microwave dielectrometry, ordering of the structure of bulk water and increasing of the amount of bound water in the presence of GS in liposome samples with a content of 10 mol% CHOL was established. (Figure 9 and Figure 10).

Finally, we demonstrated the application of liposomes composed of PC and CHOL on fibroblast cells, resulting in decreased GS toxicity (Figure 11). This highlights the usefulness of previous biophysical studies in determining the optimal liposome composition. Further extensive studies with cells are still required to determine dose-dependencies and gain deeper insights into cellular reactions to GS.

In this study, one of the aims was to elucidate the respective roles of surface charge density and hydrophobic core fluidity of lipid membranes in governing the interaction of GS with model liposomal systems. Understanding these physicochemical parameters is critical for the rational design of GS-loaded nanocarriers intended for internal drug delivery. To modulate surface charge, we employed binary mixtures of DPPC with cardiolipin—a lipid bearing two negative charges per molecule—to generate model membranes with controlled anionic lipid content. Simultaneously, to modulate membrane fluidity, we adjusted cholesterol content in DPPC-based liposomes, increasing cholesterol up to 30 mol% to systematically decrease membrane fluidity and order the acyl chains within the bilayer.

Our results reveal that membrane fluidity is the dominant factor influencing the partitioning and long-term retention of GS within the liposomal bilayer. As cholesterol content increased and membrane rigidity rose, GS incorporation into the bilayer was significantly diminished. These findings suggest that fluid membranes provide a more favorable environment for the hydrophobic interactions and lateral accommodation of GS within the lipid core. This behavior is consistent with the known amphipathic nature of GS, which tends to reside at the lipid–water interface and disturb the bilayer structure upon incorporation. More rigid bilayers may resist such perturbation, thereby limiting GS accommodation.

Conversely, the surface charge density was found to influence the initial membrane association of GS. The presence of cardiolipin facilitated electrostatic interactions between the negatively charged lipid headgroups and the cationic regions of GS, promoting early-stage binding from the aqueous phase. However, this effect plateaued and was less significant in determining steady-state GS retention, especially in membranes with reduced fluidity.

These findings carry important implications for the design of liposomal carriers for GS delivery, particularly for topical applications where localized action, retention, and minimal systemic leakage are desired. To ensure effective delivery, liposomes should possess a sufficiently fluid hydrophobic core to accommodate and retain GS, while also incorporating a modest level of anionic lipids to facilitate initial GS adsorption/uptake; therefore, a balanced cholesterol content (~30 mol%) may offer a compromise between stability and bioavailability. Based on our data, the most promising liposome formulations should include 10 mol% cardiolipin to enhance initial GS uptake and ≤30 mol% cholesterol, offering a compromise between stability and bioavailability to maintain sufficient fluidity for stable in-corporation and reduce premature release. Such lipid compositions could be used to develop GS-loaded liposomes with sustained-release properties, suitable for topical antimicrobial applications (e.g., infected wounds, dermatitis, or biofilm-associated skin infections), minimizing cytotoxic effects associated with systemic exposure.

In addition to supporting the development of GS carriers, our data may offer further therapeutic insights. Based on the observed GS interactions with CHOL-containing membranes and live cell membranes for GS targeting, we could suggest that a higher CHOL percentage could be favorable for GS binding and cell membrane disruption, potentially enhancing its therapeutic effect on CHOL-rich cells.

In conclusion, this study provides new insights into GS interactions with membranes containing CL and CHOL, which are essential for developing improved GS drug carrier formulations. Our findings not only elucidate GS–membrane interaction mechanisms but also provide a quantitative framework for optimizing liposomal delivery systems tailored for topical administration of this antimicrobial peptide.

## 4. Materials and Methods

All chemicals were purchased from Merck-Sigma-Aldrich and were of analytical grade or higher.

### 4.1. Gramicidin S-Contained Liposome Preparation

Liposome preparation is based on the method already described [30]. Shortly, L-α-dipalmitoylphosphatidylcholine (DPPC), cardiolipin (CL), and cholesterol (CHOL) were dissolved in ethanol to obtain stock solutions: 100 mg/mL of DPPC, 5 mg/mL of cardiolipin, 10 mg/mL of cholesterol. Then the components were mixed to experimentally required proportions. Removal of organic solvent residues from the lipid mixture was carried out by drying under nitrogen and then using a vacuum rotary evaporator (Eppendorf, Hamburg, Germany) for a minimum of 3 h at a temperature of 40 °C until a stable and uniform lipid film was formed. The film was fully dehydrated and produced in a reproducible manner. The lipid film was then hydrated to a suspension state with an aqueous buffer solution (Hanks’ balanced salt solution, pH = 7.4). Lipid membranes were obtained by extrusion at 25 °C by polycarbonate filter with the pore diameter of 100 nm. The final concentration of lipids in the studied liposome samples in aqueous suspension was 10 mM.

Gramicidin S (GS, PubChem CID 73357) was dissolved in ethanol and mixed into liposomes to assess final GS concentrations ranging from 0 to 80 µM. The accuracy of drying, hydration, and incubation stages was controlled by the samples’ precise weight using micro-balance Mettler XP26 (“Mettler-Toledo”, Columbus, OH, USA). The diameter of lipid vesicles was controlled by the light scattering method.

The liposome samples tested in this study varied in composition, but all contained DPPC, CL, and CHOL. Liposomes were used immediately after preparation.

### 4.2. Assessment of Dielectric Permittivity, Dielectric Losses, Dielectric Relaxation Frequency, and Static Dielectric Constant

Dielectric permittivity and dielectric losses of liposomes were measured using the microwave dielectrometry method at an operating frequency of 9.2 GHz [31,32,33]. The dielectric relaxation frequency (f_d_) and the static dielectric constant (ε_s_) were calculated using the Debye equations.

### 4.3. Membrane Fluidity Access by Fluorescence Spectroscopy

To assess membrane fluidity, we employed the lipophilic fluorescent probe pyrene, whose distribution within lipid membranes and degree of excimer formation correlate with membrane fluidity [15]. Pyrene was dissolved in ethanol at a stock concentration of 25 mg/mL immediately prior to the experiment and added to the liposome suspension at a final concentration of 15 µM. The suspension was gently mixed and incubated for 1 h at 25 °C. Excess unbound probe was washed out, and the fluorescence spectra of liposomes containing inserted pyrene were recorded using a Hitachi U-3210 spectrofluorimeter (Hitachi, Tokyo, Japan) following excitation at 319 nm and emission measurements at 390 nm and 470 nm.

### 4.4. Surface Potential Assessment by Fluorescence Spectroscopy

Surface charges of liposome membranes were examined using the water-soluble anionic fluorescent probe 8-anilino-1-naphthalenesulfonate (ANS), which integrates noncovalently into lipid structures and exhibits fluorescence that is highly sensitive to the molecular environment of the phospholipid bilayer [34]. ANS was added to the liposome suspension at a final concentration of 30 µM, the suspension was gently mixed and incubated for 10 min at 25 °C. Fluorescence was measured using a Hitachi U-3210 spectrofluorimeter (Hitachi, Tokyo, Japan) at excitation and emission wavelengths of 365 nm and 480 nm, respectively.

### 4.5. Cultivation of L929 Cell Line

L929 cells were cultured in Dulbecco’s Modified Eagle Medium/Nutrient Mixture F-12 (DMEM/F12; Biowest, Nuaillé, France), supplemented with 10% fetal bovine serum, 50 μg/mL penicillin, and 50 μg/mL streptomycin (all from Biowest, France), at 37 °C in a 5% CO_2_ atmosphere. Monolayer cultures were established in plastic culture flasks (SPL Life Sciences, Pocheon-si, Republic of Korea) starting from the seeding density of 2 × 10^5^ cells/mL.

### 4.6. Cell Treatment by GS or Liposome-Carried GS

To assess the potential cytotoxicity of liposome-carried GS, L929 cells were incubated for 24 h at 37 °C with either free GS (20 µM) [8] or GS at the same concentration encapsulated in liposomes containing 10 mol% CHOL. For this purpose, 400 µL of liposome emulsions in DMEM/F12 medium were added to each well of the plate. The plate was gently shaken for 10 min to evenly distribute the liposomes, after which the plate was transferred to a CO_2_ incubator.

### 4.7. Determination of Cell Viability

To assess the viability of cells we applied two probes, fluorescein diacetate (FDA) and ethidium bromide (EtBr) widely used in molecular biology [35,36,37,38,39,40]. FDA is a nonpolar, non-fluorescent molecule capable of crossing the plasma membrane, where it is hydrolyzed by intracellular esterases in viable cells to release fluorescent polar fluorescein. This molecule accumulates within living cells, interacting with internal components and fluorescing green at 520 nm when excited at 490 nm. In contrast, EtBr penetrates only cells with compromised membranes and binds to double-stranded nucleic acids. Its excitation and emission spectra shift upon DNA binding—from UV absorption peaks at 210 nm and 285 nm in solution to longer wavelengths (typically 300–360 nm and 485–526 nm) when bound. Free EtBr fluoresces at ~605 nm, while DNA-bound EtBr emits around 590 nm due to a slight blue shift. Stained samples were analyzed using a confocal laser scanning microscope (LSM 510 META, Carl Zeiss, Jena, Germany) at an excitation wavelength of 488 nm for FDA and 543 nm for EB. Viability was defined as the ratio of cells showing only green fluorescence to the total number of cells, multiplied by 100%, evaluating at least 100 visual fields.

### 4.8. Determination of Cell Monolayer Confluence

The confluency of the hematoxylin–eosin-stained monolayer was evaluated by scanning the bottom of the well plate with an Epson Perfection V10 scanner (Epson Europe B.V., Amsterdam, The Netherlands). The relative area occupied by the cell monolayer was quantified using AxioVision Rel 4.8 software (Carl Zeiss, Germany) and expressed as a percentage.

### 4.9. Statistical Analysis

Data from three to five independent liposome preparations are shown in the figures as means ± standard deviations (SDs). Statistical significance was determined using the Mann–Whitney test, with * indicating *p* < 0.05.

### 4.10. Data Sharing Statement

The data supporting the findings of this study are available within the article.

## 5. Conclusions

This study demonstrates that lipid-based formulations, particularly those composed of DPPC and CHOL, can effectively modulate the interaction of gramicidin S (GS) with model membranes in liposome nanocarriers, partially altering but largely preserving key membrane characteristics. Importantly, these liposomes tolerated high GS concentrations—up to 80 μM—highlighting their potential for high-dose therapeutic applications. Preliminary cytotoxicity assays further confirmed that liposomal encapsulation significantly reduces GS-induced toxicity in L929 cells. Overall, our findings support the development of rationally designed lipid nanocarriers to enhance the safety and efficacy of antimicrobial peptides like GS in biomedical contexts

## Data Availability

The original contributions presented in the study are included in the article; further inquiries can be directed to the corresponding authors.

## Abbreviations

The following abbreviations are used in this manuscript:

GS	Gramicidin S
AMP	Antimicrobial peptide
PC	Phosphatidylcholine
DPPC	Dipalmitoylphosphatidylcholine
DLS	Dynamic light scattering
CL	Cardiolipin
CHOL	Cholesterol
